# Cobalt Stabilization through Mesopore Confinement on TiO_2_ Support for Fischer–Tropsch Reaction

**DOI:** 10.1021/acsaem.3c01432

**Published:** 2023-09-08

**Authors:** F. Platero, S. Todorova, L. Aoudjera, L. Michelin, B. Lebeau, J. L. Blin, J. P. Holgado, A. Caballero, G. Colón

**Affiliations:** † Instituto de Ciencia de Materiales de Sevilla, Centro Mixto Universidad de Sevilla-CSIC, Américo Vespucio, 49, 41092 Sevilla, Spain; ‡ Institute of Catalysis, Bulgarian Academy of Sciences, 1113 Sofia, Bulgaria; § Université de Lorraine/CNRS, L2CM, UMR7053, 54500 Vandoeuvre-lès-Nancy, France; ∥ Université de Haute Alsace, CNRS, IS2M UMR 7361, 68100 Mulhouse, France; ⊥ Université de Strasbourg, 67000 Strasbourg, France

**Keywords:** Cobalt, TiO_2_, mesostructured, SMSI, Fischer−Tropsch

## Abstract

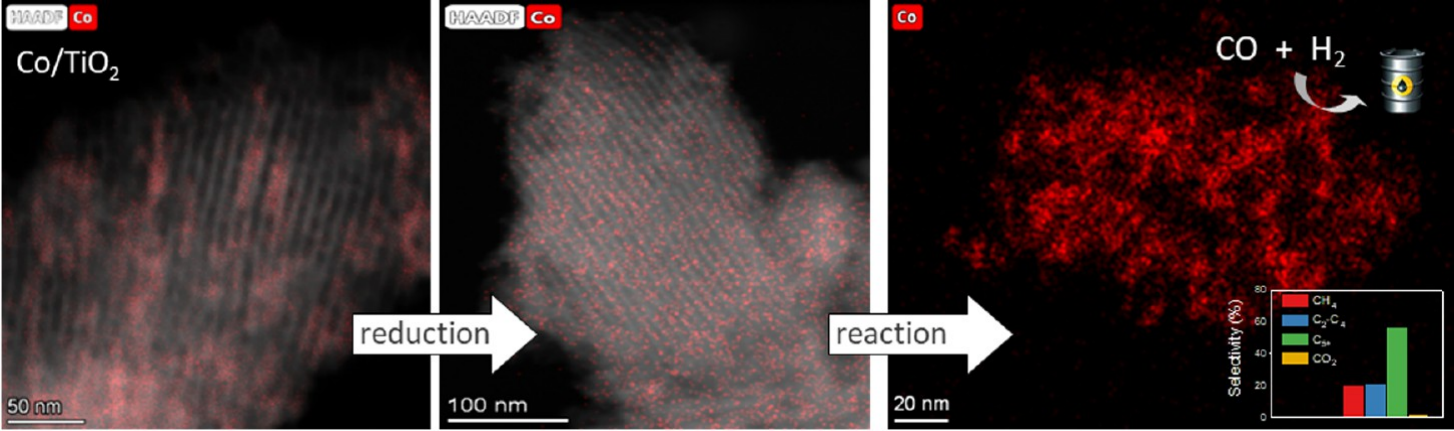

Cobalt supported on mesostructured TiO_2_ catalysts has been prepared by a wet-impregnation method. The Co/TiO_2_ catalytic system showed better catalytic performance after support calcination at 380 °C. Co nanoparticles appeared well distributed along the mesopore channels of TiO_2_. After reduction pretreatment and reaction, a drastic structural change leads to mesopore structure collapse and the dispersion of the Co nanoparticles on the external surface. Along this complex process, Co species first form discrete nanoparticles inside the pore and then diffuse out as the pore collapses. Through this confinement, a strong metal–support interaction effect is hindered, and highly stable metal active sites lead to better performance for Fischer–Tropsch synthesis reaction toward C_5+_ products.

## 1 Introduction

Energy underlies all the major economic changes that have occurred throughout history, especially those of the two last centuries. We are undoubtedly facing a train wreck in the actual energy scenario, brought on by the combination of the ambitious aim to deplete fossil fuels and, on the other side of the balance, the progressive increase in energy demand due to population increase and quality of life.^1^ Within this framework, less carbon-intensive alternatives are trying to progressively replace traditional fuels. Gas-to-liquids (GtL) and coal-to-liquids (CtL) technologies are becoming important and fundamental strategies.^2,3^ Within these processes, CO and CO_2_ turned novel raw materials to produce fuels and have attracted much interest in the last years.^4−6^

Among these processes involving CO and CO_2_, Fischer–Tropsch synthesis (FTS) is one of the most desired catalytic processes for the production of clean hydrocarbon fuels. Thus, from the mixture of CO and H_2_ (derived from coal, biomass, natural gas, or waste), environmentally responsive fuels and chemicals can be obtained.^7,8^ For industrial applications, Co-based TiO_2_ catalysts appear particularly attractive, giving high activity performance and interesting selectivity values to targeted C_5+_ liquid hydrocarbons.

In spite of this, we have to say that TiO_2_-supported cobalt catalysts have been less investigated than those comprising SiO_2_ or Al_2_O_3_. This can be probably related to the higher complexity of the Co/TiO_2_ systems.^9,10^ Thus, the particular structural features of titania with different crystallographic polymorphs (anatase, rutile, brookite) as well as the occurrence of the strong metal–support interaction (SMSI) could be the origin of the lower impact in the literature. From a practical point of view, the main challenges for the improvement of FTS catalysts are related to achieving higher activity, improved selectivity to targeted product (long-chain hydrocarbons or light olefins), and improved catalyst lifetime. Within this frame, it has been reported that the interaction between metal nanoparticles and the support could imply both, an emerging tool in catalyst design with enhanced performance but also a challenge when this interaction is detrimental.^11,12^ As widely discussed, this interaction occurs when the support is partially reduced at the vicinities of the metal nanoparticle, leading to the generation of mobile suboxide species (e.g., TiO_*x*_, NbO_*x*_) that would cover in part the surface of the metal nanoparticle.^13^ For the Fischer–Tropsch reaction, reducible oxidic supports have a major influence on the catalyst performance.

In a previous work, we have stated that cobalt deposition over APTES functionalized TiO_2_ leads to well-dispersed Co nanoparticles on the TiO_2_ surface. Moreover, we demonstrated that due to the close interaction with silicon, cobalt metallic sites would be protected against SMSI and even from catalyst deactivation, avoiding the formation of surface inactive species.^14^ In the present study, we tackle the same objective of Co stabilization but through a different strategy in this case. The deposition of cobalt on a mesostructured TiO_2_ leads to the formation of well-dispersed Co clusters confined on the mesoporous structure that would constrain the Co particle size. Through a wide characterization, we have demonstrated that this starting structural situation strongly conditions the behavior of Co species during reduction and reaction steps and completely hinders the SMSI effect.

## 2 Experimental Section

### 2.1 Synthesis of Co/TiO_2_ Catalysts

**Figure 1:**
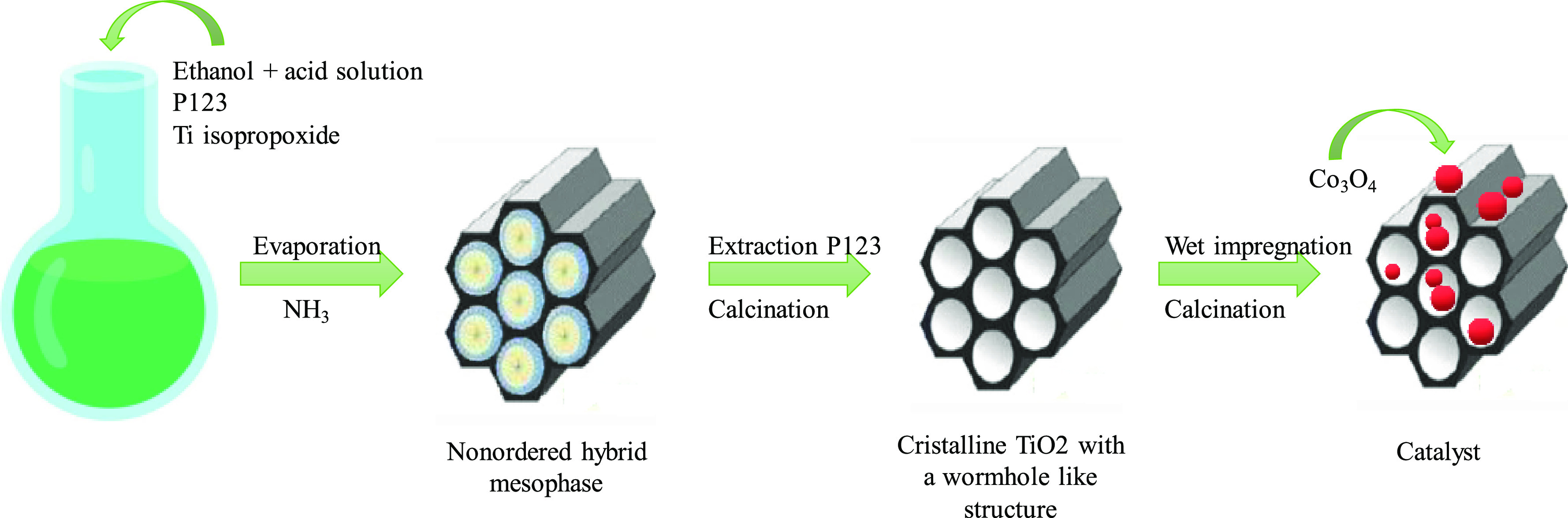
Schematic representation of the synthesis route used to prepare the catalysts of cobalt on TiO_2_ mesoporous.

Mesoporous titania was prepared by the surfactant templating pathway using triblock copolymer P123 (EO)20(PO)70(EO)20 (EO = ethylene oxide, PO = propylene oxide) (Sigma-Aldrich) as a structure-directing agent (Figure 1). First, 1 g of surfactant was dissolved in 20 g of ethanol (absolute, VWR) under stirring at room temperature. Then, 2 g of a hydrochloric acid solution (ACS reagent 37%, Sigma-Aldrich) and 3 g of titanium isopropoxide (Ti(iPrO)_4_, 97%, Sigma-Aldrich) were added to that solution. The mixture was directly evaporated under vacuum (55 °C, 25 mbar) to remove ethanol and 2-propanol released by hydrolysis of Ti(iPrO)_4_. The obtained solid was dried in an oven at 40 °C for 12 h and subsequently placed under an atmosphere of NH_3_ for 12 h to allow the condensation of the inorganic precursor. The final product was recovered after surfactant extraction with ethanol by means of a Soxhlet method during 8 h.^15^ Thus, the obtained powder would be an almost free surfactant amorphous TiO_2_ precursor. Finally, this solid was calcined at 3 different temperatures (300, 340, and 380 °C) in order to study the effect of the calcination temperature of the support on the performance of the catalytic systems.

A cobalt metallic phase was deposited over synthesized supports through the incipient wetness impregnation method. All supported catalysts were prepared with a nominal loading of 10 wt % Co, using Co(NO_3_)_2_·6H_2_O (Sigma-Aldrich) as the metal precursor. Then, the catalytic system was dried at 80 °C for 2 h and calcined in flowing air at 250 °C for 2 h. A reference catalyst was prepared by the same method using a commercial TiO_2_ (Evonik P90) support in order to compare its performance with that of the mesoporous supports. The catalysts were denoted as Co/TiO_2_-300, Co/TiO_2_-340, Co/TiO_2_-380, and Co/P90.

### 2.2 Characterization Techniques

The cobalt content in the catalysts was determined by inductively coupled plasma atomic emission spectroscopy (ICP-OES) using an iCAP 7200 Duo spectrometer. Solids were previously dissolved by using the microwave digester Ethos Easy.

Surface analysis by N_2_ adsorption/desorption isotherm measurements was carried using a Micromeritics Tristar II instrument. Specific surface areas were calculated according to the BET method while pore size distribution was determined by the BJH method.

SAXS measurements were carried out using a SAXSess mc2 (Anton Paar) instrument. attached to an ID 3003 laboratory X-ray generator (General Electric). Sealed X-ray tubes (PANalytical, λCu (Kα) = 0.1542 nm) operating at 40 kV and 50 mA were used. A translucent beam-stop allowed the measurement of an attenuated primary beam at *q* = 0. Mesoporous catalysts were introduced into a powder cell inside a chamber equipped with a temperature-controlled sample holder unit. X-ray scattered beams were recorded by a CCD detector placed 309 mm from the sample holder in the *q* range from 0.09 to 5 nm^–1^. All data were corrected for the background scattering from the empty cells.

Wide angle X-ray powder diffraction (XRD) of catalysts was obtained by a Siemens D-501 diffractometer with a Ni filter and graphite monochromator and using Cu Kα radiation. The data were acquired in a 2θ range of 10°–90°, setting a step of 0.05° and an acquisition time of 160 s.

Micro-Raman measurements were performed by using a LabRAM Jobin Yvon spectrometer equipped with a microscope. Laser radiation (λ = 532 nm) was used as the excitation source at 5 mW. All measurements were recorded under the same conditions (2 s of integration time and 30 accumulations) using a 100× magnification objective and a 125 mm pinhole.

The reduction profile of the Co/TiO_2_ catalysts was studied by H_2_-TPR analyses using a Quantachrome Chemstar instrument equipped with a TCD and a mass spectrometer. Before each experiment, 30 mg of catalyst was first degassed at 150 °C for 30 min under an inert flow of Ar at 25 mL/min. Then, the analysis was carried out from room temperature up to 900 °C at a heating ramp of 10 °C/min, by fluxing H_2_(5%)/Ar at 10 mL/min.

Transmission electron microscopy (TEM) images and high angle annular dark field (HAADF) and element mapping analysis images were obtained by the equipment FEI S/TEM Talos F200S. Samples were prepared by dipping a carbon grid in the powder sample.

X-ray photoelectron spectroscopy (XPS) experiments were carried out on VG-escalab 210 equipment over pelletized samples. Samples were introduced in a prechamber at 10^–7^ Torr. Acquisition was performed in an appendant analysis chamber equipped with a SPECS Phoibos 100 hemispheric analyzer at 10^–9^ Torr using Mg Kα radiation (*E* = 1.5418 keV) with 20 mA of anode current and 12 kV of potential acceleration. A Ti*2p* signal (458.5 eV) was used as the internal energy reference in all the experiments.

### 2.3 Catalytic Activity in Fischer–Tropsch Synthesis Reaction

For the catalytic studies, we used 0.25 g of catalyst diluted in 0.25 g of SiC that was placed in a stainless-steel fixed-bed tubular reactor. Previous to the reaction, catalysts were *in situ* reduced by flowing H_2_ (50%) in N_2_ at 260 °C for 13 h. Then, the pressure was set to 1.0 MPa, and finally, CO was introduced. Reaction steam consists of a CO/H_2_/N_2_ flow with a volume ratio of 1:2:2 (N_2_ was used as internal standard for gas chromatography analysis) and a flow rate of 35 mL/min through the reactor which gives a constant gas hourly space velocity (GHSV) of 4200 h^–1^ considering the catalyst bed volume. The reaction was followed for 6 h until reaching the pseudosteady state. Heavier hydrocarbons traces were condensed in a trap located at the reactor outlet and kept at 100 °C. Reaction products were analyzed by means of a previously calibrated GC (Agilent 7820) equipped with TCD and FID detectors. All tubing pipes from the reactor to the GC were thermostated to prevent the condensation of products.

Conversion and selectivity values were calculated using the following equations:
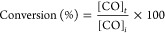
1where [CO]_*t*_ represents the moles of reacted carbon monoxide, and [CO]_*i*_ the initial carbon monoxide amount.
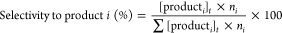
2where [product_*i*_]_*t*_ represents the moles of the specific product, *n*_*i*_ is the number of C atoms in the product molecule, and Σ[product]_*i*_ is the sum of moles from all products in the reaction. Product selectivities are expressed on a carbon basis.

## 3 Results and Discussion

### 3.1 Structural and Textural Properties

**Figure 2:**
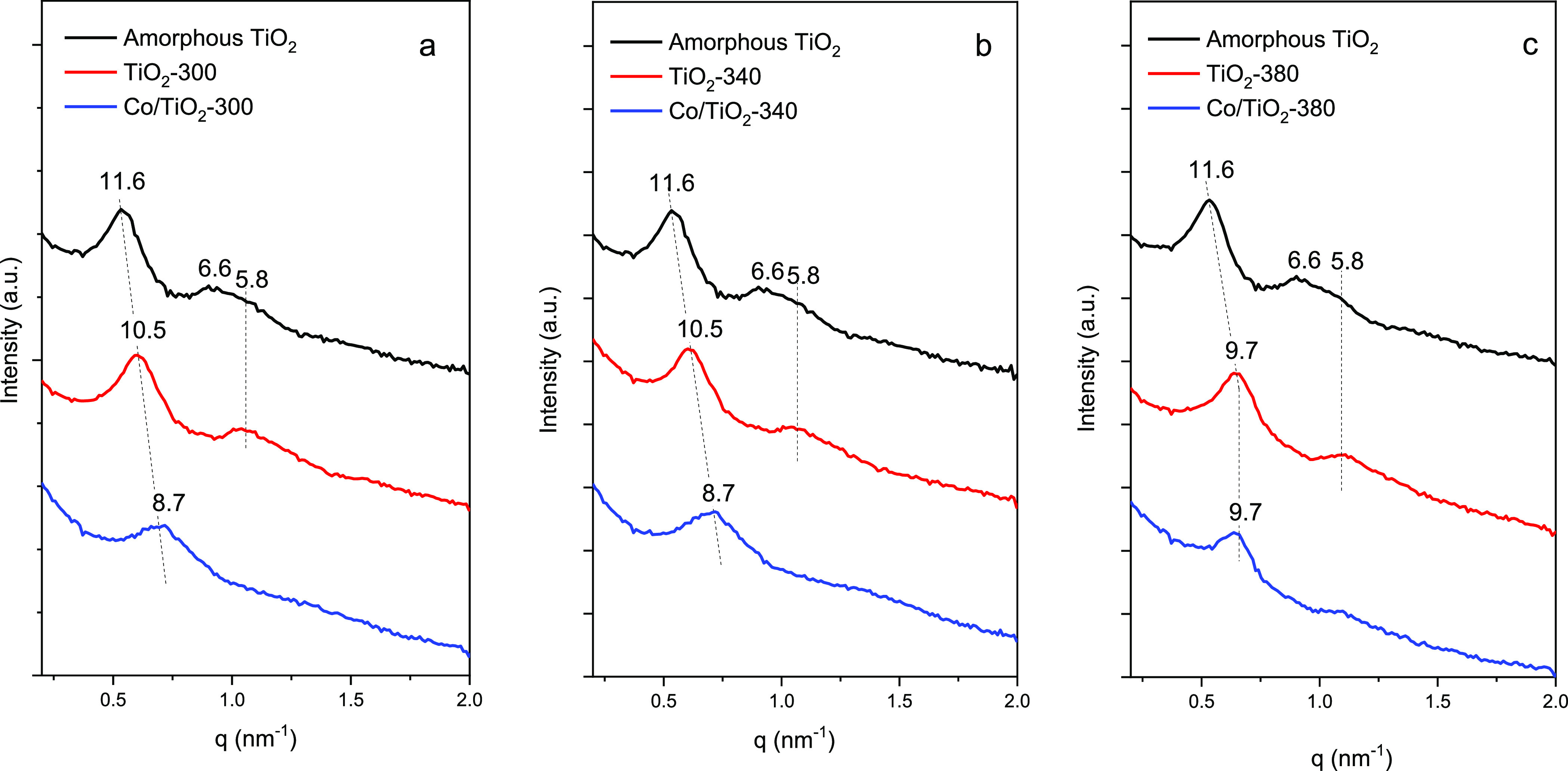
SAXS patterns of amorphous TiO_2_ (black line), pristine TiO_2_ obtained after calcination (red line), and Co/TiO_2_ (blue line) for (a) TiO_2_-300, (b) TiO_2_-340, and (c) TiO_2_-380.

The SAXS patterns of the TiO_2_ supports and calcined Co/TiO_2_ catalysts are shown in Figure 2. The amorphous TiO_2_ exhibits three reflections at 11.6, 6.6, and 5.8 nm. After calcination, two clear peaks remain corresponding to the relative position of the (10) and (20) Bragg reflections attributed to the 2D-hexagonal mesostructure of the *P6m* space group.^16,17^ It is worth noting that when calcining at 380 °C, a shift in the position of the first reflection toward a lower *d*_100_ value is observed (Table 1). This phenomenon can be attributed to a condensation of the Ti–OH groups but also to the partial crystallization of the walls.^18^ The cell parameter *a*_0_ can be calculated from the relation *a*_*0*_ = 2*d*_100_/ giving a value of 12.1 nm for TiO_2_ calcined at 300 and 340 °C and 11.2 nm after calcination at 380 °C. Such a decrease would denote a certain contraction of the mesoporous network.

**Table 1:** Structural and Surface Features of TiO_2_ and Co/TiO_2_ Materials

Sample	*d*_100_ (nm)^a^	BET (m^2^/g)	*D*_p_ (nm)	*V*_p_ (cm^3^/g)
TiO_2_ uncalcined	11.6	384	9.2	0.62
TiO_2_-300	10.5	265	8.6	0.47
TiO_2_-340	10.5	281	8.3	0.50
TiO_2_-380	9.7	228	7.0	0.44
Co/TiO_2_-300	8.7	200	8.2	0.37
Co/TiO_2_-340	8.7	219	8.3	0.40
Co/TiO_2_-380	9.7	170	6.9	0.32
Co/P90	—	75	3 + 30	0.23

a *d*_100_ spacing values were calculated from the first SAXS peak using *d*_100_= 2·π/*q*.

After Co impregnation and subsequent calcination at 250 °C, the secondary reflections become less resolved, denoting a less ordered mesoporous network. In addition, there is a greater contraction of the mesopore network for calcined TiO_2_ at low and medium temperatures, giving *d*_100_ values of 8.7 nm (Table 1).

However, the support calcined at 380 °C does not present changes in its network dimension with respect to unsupported TiO_2_ once the metallic phase is added.

**Figure 3:**
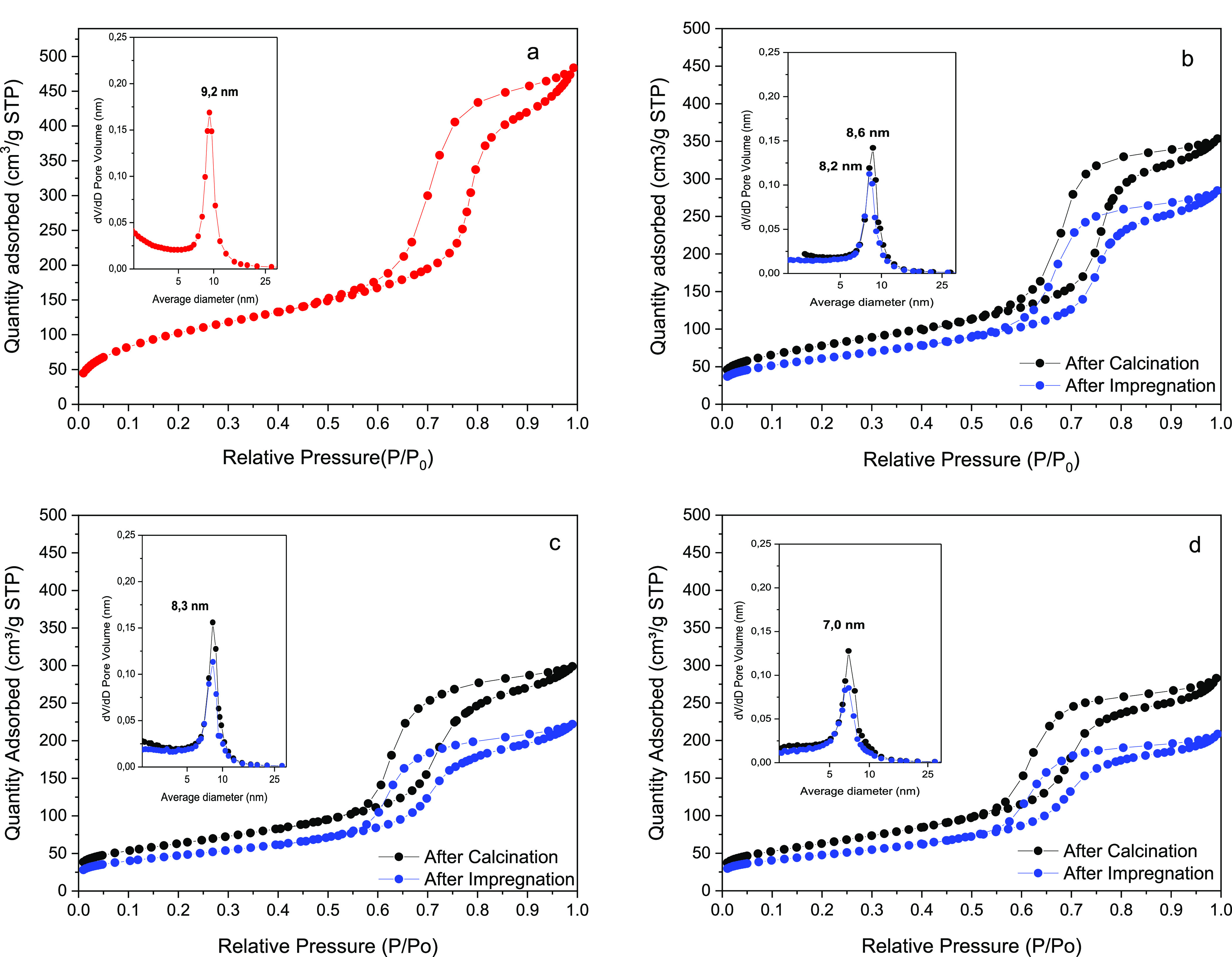
Nitrogen adsorption–desorption isotherm with the corresponding pore size distribution for (a) amorphous TiO_2_, (b) TiO_2_-300, (c) TiO_2_-340, and (d) TiO_2_-380.

The adsorption isotherms and the corresponding pore size distribution obtained by N_2_ physisorption are shown in Figure 3. The textural properties of the obtained mesoporous materials are summarized in Table 1. All systems present a type IV adsorption isotherm, which is characteristic of mesoporous solids. Thus, the presence of the hysteresis loop is clearly associated with typical capillary condensation during adsorption/desorption inside mesopores.^19^ After the organic phase extraction process with the solvent, amorphous TiO_2_ exhibits a high specific surface area (BET) of 384 m^2^/g.

Further calcination of the TiO_2_ precursor solid significantly affects the specific surface area, which exhibits a noticeable decrease with calcination temperature (Table 1). This fact is related to the contraction of the mesoporous network, as already stated by SAXS.

Thus, as the calcination temperature increases, both surface and crystalline features appear notably affected. Once cobalt was deposited with the subsequent calcination process, the BET surface area showed an additional diminution.

This second loss of surface area may be associated with the introduction of cobalt particles into the mesoporous channel, being significantly important for Co/TiO_2_-380. Even so, the final obtained cobalt-supported TiO_2_ catalysts still show high surfaces, between 219 and 170 m^2^/g.

Regarding the mesoporous structure, in all cases, a homogeneous and well-defined pore size distribution is observed (insets in Figure 3). A decrease in the *dV/dD* values is observed after the calcination process and after cobalt incorporation. Such a decrease could be caused by the presence of cobalt species inside the pores of the mesoporous network, or even to organic molecules from the synthesis process, which would partially block the pores of the material.^20^ It is also worth mentioning that the average pore size decreased after the calcination treatment of the supports, being more evident in the solid calcined at 380 °C, going from 9.2 to 7 nm. This fact agrees with the greatest contraction of the mesoporous network observed by SAXS.

**Figure 4:**
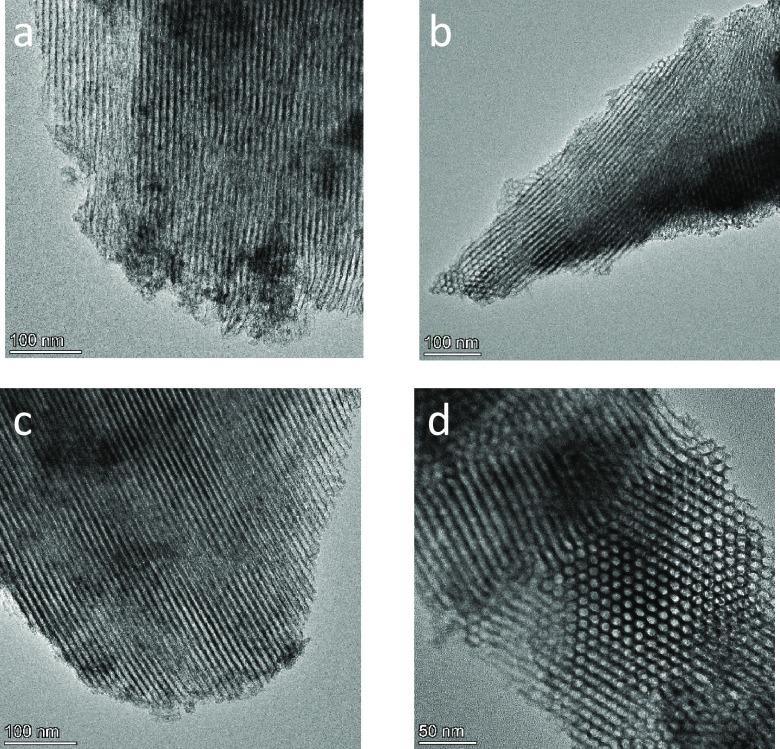
TEM images for (a) TiO_2_-300, (b) TiO_2_-340, and (c, d) TiO_2_-380 supports.

The mesopore ordering already stated can be clearly observed by TEM (Figure 4). Thus, it can be confirmed that the presence of a more defined mesoporous structure becomes obvious as the calcination temperature of the solid increases.

**Figure 5:**
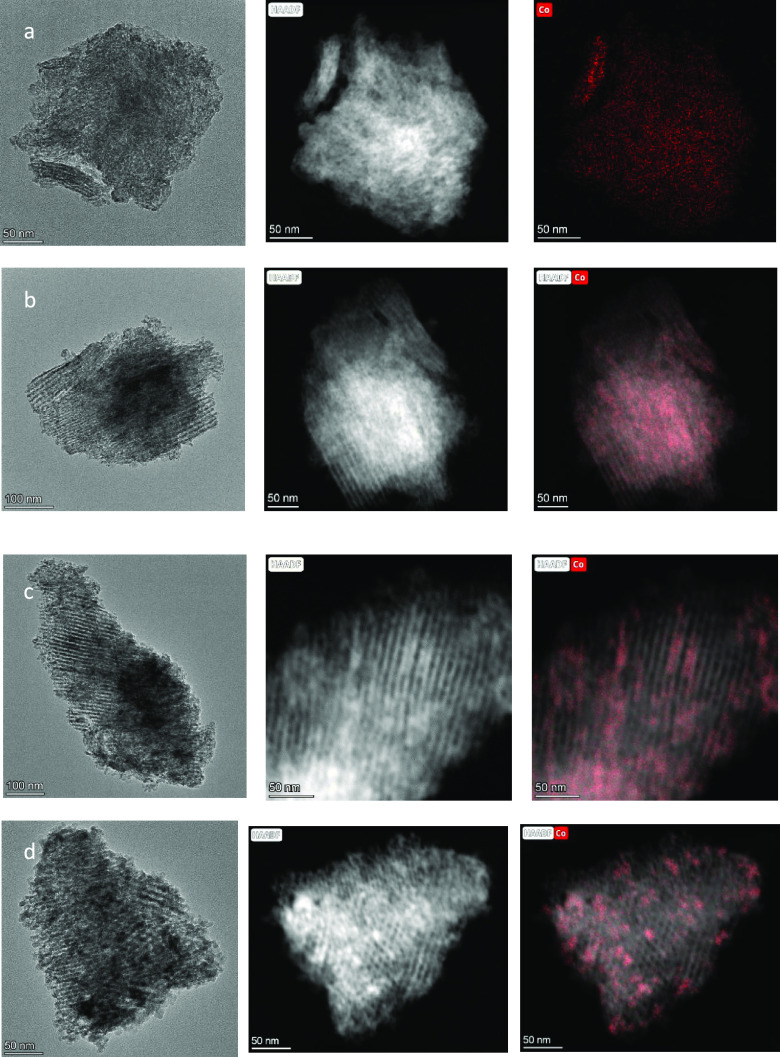
HAADF-STEM images of the Co/TiO_2_-300 (a), Co/TiO_2_-340 (b), and Co/TiO_2_-380 (c, d) catalysts.

The HAADF-STEM images of the cobalt-impregnated systems also confirm less order of the mesopore network after cobalt addition (Figure 5). This effect is much more notable in the support calcined at a lower temperature. On the contrary, the TiO_2_ support calcined at 380 °C practically shows its mesoporous network unaffected after cobalt impregnation.

It is also worth mentioning the evolution of the cobalt disposition on different TiO_2_ supports. The HAADF-STEM images and EDX Co element mapping for the Co/TiO_2_-300 catalyst show a dispersion of the cobalt particles throughout the surface of the support, which practically does not present a mesoporous structure (Figure 5a). In the Co/TiO_2_-340 system, cobalt seems to be partially located inside the mesoporous channels. However, a fraction of Co appears agglomerated, where the mesoporous structure is less defined (Figure 5b). For Co/TiO_2_-380, though in some areas it is possible to observe certain Co agglomeration, in general it is observed that cobalt is spread located inside the channels of the mesoporous structure (Figure 5c, d).

Therefore, it can be envisaged that cobalt diffusion along the mesoporous network is favored by the calcination temperature of the support. As stated before, higher temperature leads to a certain pore channel contraction, accompanied by the loss of remaining rest of the organic template and more definition of the porous network that would favor Co species diffusion.

**Figure 6:**
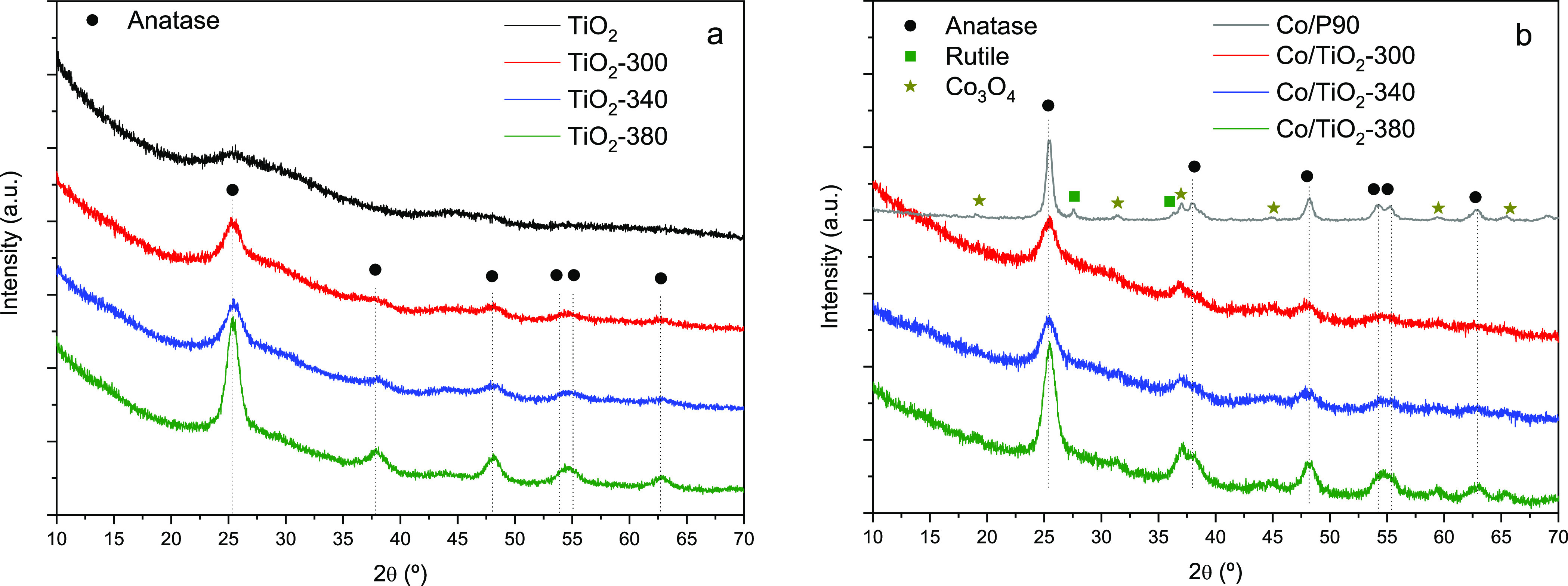
X-ray diffraction patterns for (a) mesoporous TiO_2_ supports and (b) Co supported catalysts.

As stated, the distribution of the metallic phase in these mesoporous systems is quite different from that observed in commercial support TiO-P90, where cobalt particles with a range of heterogeneous sizes between 15 and 20 nm have been observed (Figure S1). The diffractograms obtained by wide angle XRD analysis are shown in Figure 6. For prepared TiO_2_ before calcination, the XRD pattern shows a very broad peak around 25°, that could be associated with amorphous TiO_2_.^21,22^ Once the amorphous solid is calcined, the XRD profile develops toward the characteristic diffraction pattern of the anatase phase. No reflections associated with other TiO_2_ phases such as rutile are observed.

As the calcination temperature of the TiO_2_ support increases, the XRD peaks become more intense and defined. This effect is related to the greater crystallinity of the anatase phase in the calcined support at higher temperature. This fact is in line with what has been previously observed by SAXS.

No changes were observed in the anatase phase of the supports after the cobalt was added. Even more, the presence of the Co_3_O_4_ or CoO phase is hardly observed probably due to the high dispersion and low size of Co NP as stated from TEM images. Though with a very poor definition, the presence in the catalytic systems of the anatase and cobalt spinel phases was confirmed by Raman spectroscopy (Figure S2). For the reference system, the anatase phase is mostly observed, with a small fraction of the rutile phase. In the case of the Co/P90 catalyst, the occurrence of Co_3_O_4_ species can be also envisaged from XRD.

### 3.2 Reducibility and Surface Properties

**Figure 7:**
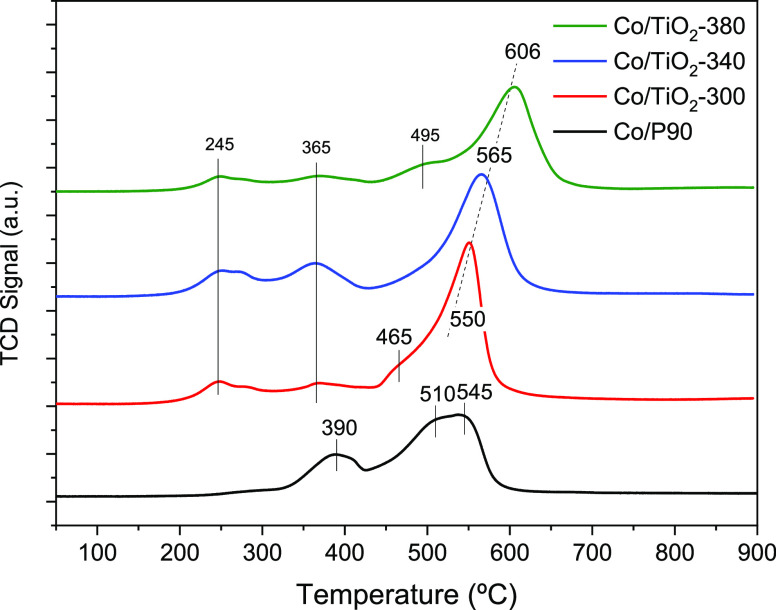
H_2_-TPR profiles for Co/P90 and Co/TiO_2_ catalysts.

In order to study the reducibility of the Co-based catalytic systems, H_2_-TPR was performed (Figure 7). Thus, reference system Co/P90 shows a bimodal profile pointing out the occurrence of two reduction processes at ca. 390 and 520 °C, which can be respectively associated with the well-known two-step reduction of Co_3_O_4_ to Co^0^ (Co_3_O_4_ → CoO; CoO → Co^0^).^23^

On the other hand, for the mesostructured system, two clear changes are observed in the reduction profile with respect to the Co/P90 catalyst. First, the lower temperature reduction is divided into two different reduction processes. Both processes, located at 245 and 365 °C, appear shifted toward a lower temperature with respect to that observed in the commercial catalyst (ca. 390 °C). These reductions observed for Co/TiO_2_ would be associated with surface cobalt particles that appear to be more dispersed than in the commercial catalyst, that clearly shows lower reduction temperature. On the other hand, the peak associated with the main reduction process shows a shift toward higher temperatures in mesostructured systems. In addition, it can be observed that such displacement is larger as the calcination temperature of the support increases.

The reduction process at higher temperatures could be associated with cobalt particles in strong interaction with the support. In our case, such a strong interaction between cobalt and the TiO_2_ support would correspond to Co species dispersed along the channels of the mesoporous network. Therefore, the observed modifications in the H_2_-TPR for Co/TiO_2_ could be correlated to the different Co distributions observed by HAADF (Figure 5). So, from the TPR study, we may corroborate a stronger Co interaction with the support and a better dispersion inside the channels observed for Co/TiO_2_-380 (Figure 5c).

Furthermore, the formation of cobalt titanate species (CoTiO_3_) would be discarded since it has been reported that this species would reduce at temperatures above 700 °C.^24,25^

**Figure 8:**
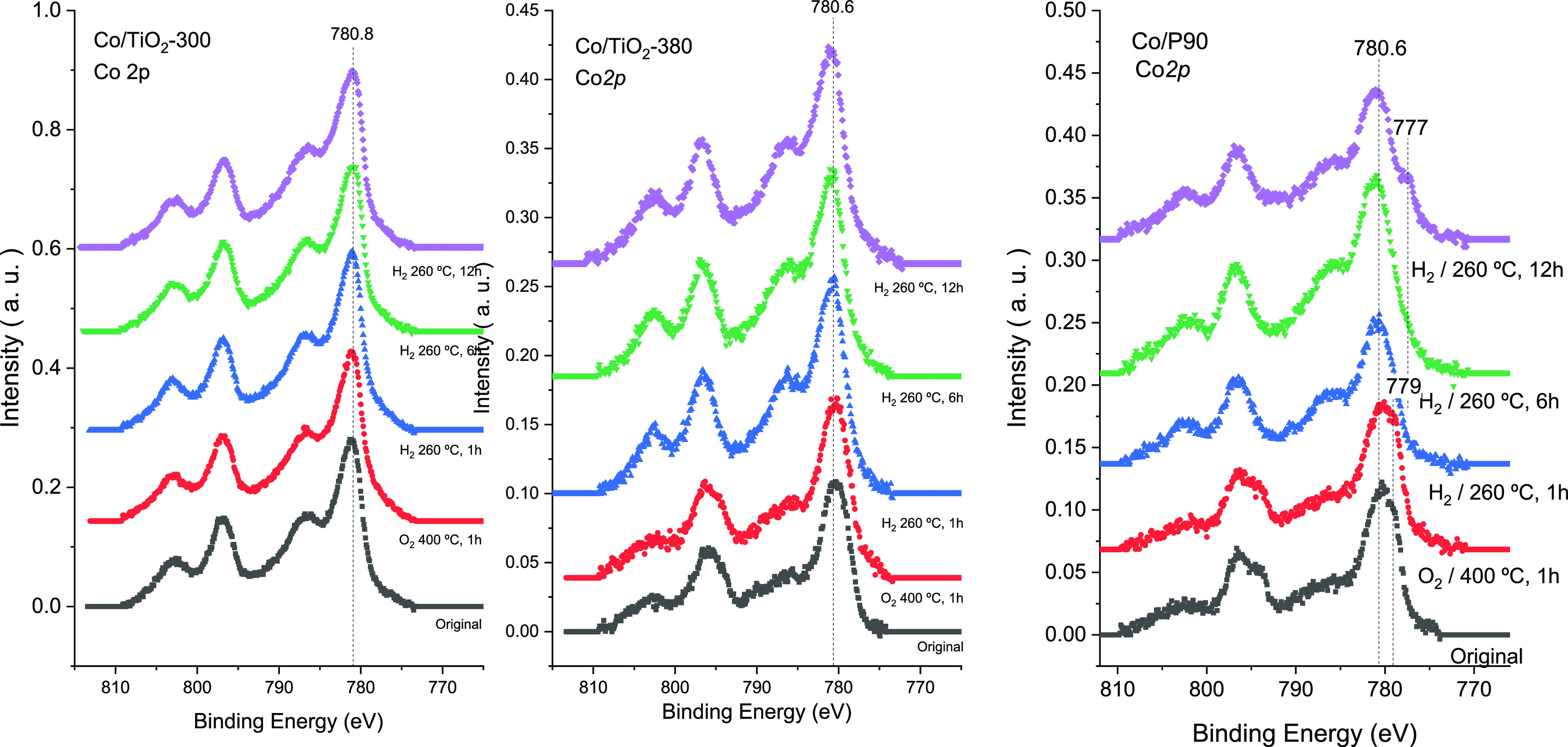
Evolution of the Co*2p* signal from XPS analysis during reduction treatment for Co/P90 and Co/TiO_2_ catalysts.

The surface features of cobalt in the catalytic systems have been determined by XPS. In Figure 8, we have depicted the Co*2p* signal from XPS analysis for mesoporous Co/TiO_2_-supported catalysts and also for the Co/P90 reference catalyst during *in situ* reduction treatment. As can be seen, the fresh Co*2p* curve for Co/P90 can be decomposed in two contributions centered at binding energies of 779 and 780.6 eV that could be ascribed respectively to Co^3+^ (corresponding to spinel Co_3_O_4_) and Co^2+^ (associated with CoO as well as Co_3_O_4_).^26−29^ This Co distribution was already discussed by us in a previous work.^12^ In that case, we estimated 68% of the spinel content from the relative areas of deconvoluted peaks. However, for Co-supported mesostructured TiO_2_ only Co^2+^ can be envisaged, showing only the contribution at ca. 780 eV. This is a first important consideration that clear differences both systems.

Furthermore, the Co/TiO_2_-300 and Co/TiO_2_-380 systems do not show any indication of Co reduction upon H_2_ treatment for 12 h. Thus, we may infer that surface Co species would remain unreduced after 12 h of H_2_ treatment at 260 °C. On the other side, Co/P90 clearly shows an incipient contribution on Co*2p* at 777 eV that denotes the progressive reduction after 6 h (Figure 8). Therefore, it can be argued that Co deposition over mesostructured TiO_2_ systems significantly conditions the chemical state of surface Co species. Unlike Co/P90, for mesostructured Co/TiO_2_, only Co^2+^ is envisaged that would not be reduced after long time reduction treatment at 260 °C.

By comparison of Co/TiO_2_-300 and Co/TiO_2_-380, it is possible to see another important issue. Surface cobalt content in Co/TiO_2_-300 is much higher than that obtained for Co/TiO_2_-380 (Table 2). If we consider the morphological features of both Co/TiO_2_ systems (Figure 5), it is clear that in the case of TiO_2_-300 cobalt is deposited widespread. Unlike for TiO_2_-380, we observed that cobalt deposition takes place preferentially in the inner mesopore network. Probably, though, a mesoporous network is formed in TiO_2_-300, the lower calcination temperature is not sufficient to completely eliminate the organic rest from structure-directing template hindering the diffusion of Co inside the pores during impregnation. Additionally, for the Co/TiO_2_-380 system, we can observe that the Co/Ti ratio progressively increases during reduction treatment (ca. 20% increases after 12 h reduction with respect to the fresh system). Thus, it is worth noting that upon reduction surface cobalt species would diffuse and enhance the dispersion of Co clusters, leading to a higher XPS signal. Such Co species would remain unreduced. For Co/P90, the calculated Co/Ti ratios are similar to those observed for Co/TiO_2_-380. In this case, since all Co should be located at the surface, the lower Co/Ti would point out a higher Co NP size (Figure S1).

**Table 2:** Calculated Co/Ti Ratios from XPS Analysis of Co/P90 and Mesoporous Co/TiO_2_ Systems^a^

Treatment	Co/TiO_2_-300	Co/TiO_2_-380	Co/P90
Original	0.162 (−8%)	0.075 (−1%)	0.067
400 °C/O_2_, 1 h	0.176	0.076	0.069
260 °C/H_2_, 1 h	0.173 (−2%)	0.085 (+12%)	0.075
260 °C/H_2_, 6 h	0.166 (−6%)	0.087 (+14%)	0.098
260 °C/H_2_, 12 h	0.187 (+6%)	0.092 (+21%)	0.083

a in parentheses we indicate the increase in Co/Ti ratio with respect to the system calcined at 400 °C in O_2_.

From the H_2_-TPR analysis after reduction treatment at 260 °C for 12 h (not shown), we have calculated that ca. 80% of Co is reduced in mesostructured Co/TiO_2_-380 vs 45% found for Co/P90.^14^ Therefore, we may say that in the case of mesostructured systems reduction treatment affects exclusively inner cobalt while surface Co would remain oxidized.

**Figure 9:**
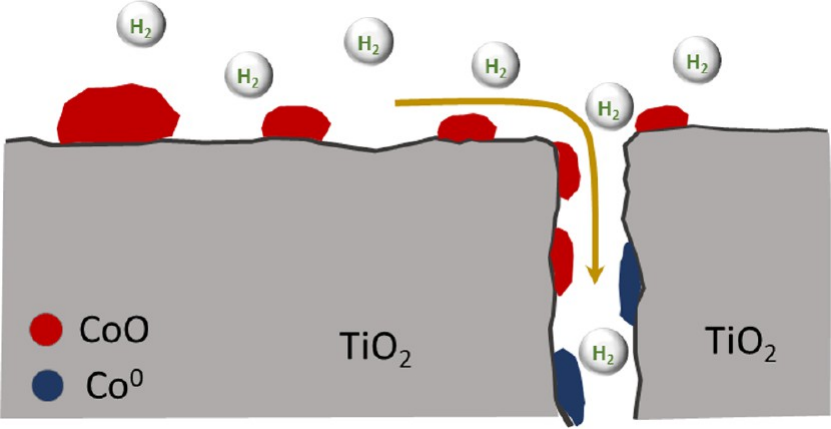
Co evolution during reduction treatment on the Co/TiO_2_-380 catalyst.

In order to understand the behavior of Co clusters during the reduction step, we have performed H_2_-TPR experiments after reduction at 260 °C for 13 h (Figure S3). From these TPR experiments, it is possible to assess the amount of reduced Co during reduction treatment. Thus, in the case of Co/P90, we have calculated that 45% of Co is reduced. Furthermore, remaining unreduced Co species show a subsequent TPR reduction peak at lower temperature (ca. 450 °C). This slight diminution in the reduction temperature would denote a change in the metal structuration on the support. On the other hand, Co/TiO_2_-380 clearly shows a much higher reduction degree (ca. 80% of the Co). As in the case of Co/P90, the reduction temperature of the unreduced Co appears at a lower temperature around 450 °C. This result seems to be in contradiction to that obtained from XPS. As discussed above from XPS results, for the Co-TiO_2_-380 catalyst, the surface Co seems to remain unreduced during the reduction treatment (Figure 8). Furthermore, the higher Co/Ti values obtained would point out a higher dispersion of surface Co or an increase in surface Co species. Since most of the Co appeared reduced from TPR (Figure S3), the lower Co/Ti would indicate a higher dispersion of surface Co species. Combining both results, we may infer that during reduction treatment Co/P90 and Co/TiO_2_ catalysts follow different pathways conditioned by the surface feature of the support. Thus, in the case of Co/TiO_2_-380, Co species would diffuse inside the pores, where they get reduced (Figure 9). This mechanism would explain the absence of surface Co^0^ and lower Co/Ti. Such metal evolution and structuration inside the mesostructure channels would also prevent the SMSI effect.

### 3.3 Catalytic Studies for Fischer–Tropsch Synthesis Reaction

**Figure 10:**
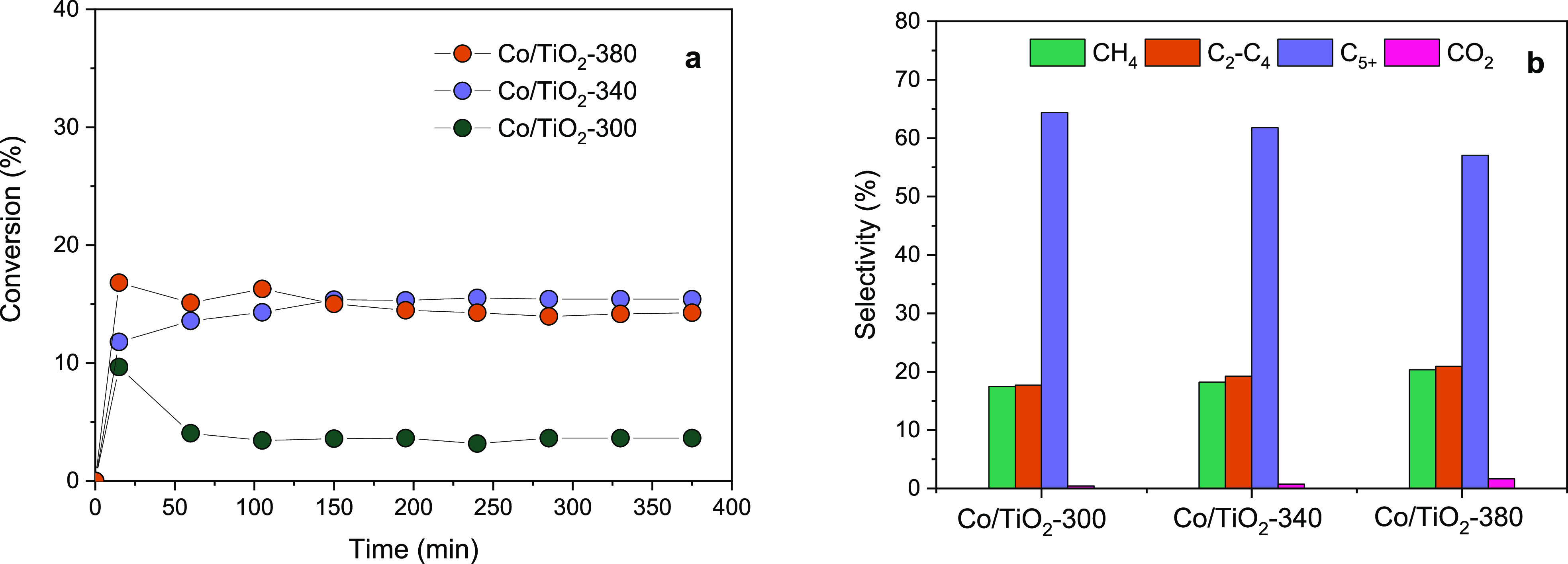
(a) CO conversion rates and (b) products selectivity in FTS reaction. Constant reaction conditions: 0.25 g of catalyst diluted in 0.25 g of SiC, *T* = 260 °C *P* = 1 MPa, GHSV = 4200 h^–1^, CO:H_2_:N_2_ = 1:2:2.

The catalytic activity of the mesostructured systems is shown in Figure 10. CO conversion rates appear higher as the calcination temperature of the mesostructured TiO_2_ support increases above 300 °C. It is worth noting that the Co/TiO_2_-300 catalyst shows a drastic initial decay in the conversion, reaching a steady-state rate below 5%. This scarce performance could be related to the low calcination temperature of the support that could surface and structurally evolve during the first reaction period. On the other hand, a CO conversion rate of ca. 15% is attained for Co/TiO_2_-340 and Co/TiO_2_-380. For these catalysts, a very stable conversion rate is attained, reaching a pseudosteady state just from the first stage of reaction. We have to remind that the Co/TiO_2_-380 catalyst showed a better-defined mesoporous structure with cobalt highly dispersed along the channels. Furthermore, during reduction pretreatment, a fraction of Co species initially located at the surface clearly diffused to the internal surface. As we have discussed, this behavior is not observed for Co/TiO_2_-300. This fact would explain the observed deactivation exclusively in this system.

Regarding the product distribution obtained, it can be pointed out that the attained selectivities to C_5+_ for all catalysts show outstanding values at around 60%, slightly decaying from 65% to ca. 60% as calcination temperature increases. At the same time, a slight increase in methane and C_2_–C_4_ formation at the expense of heavy hydrocarbons is observed as the calcination temperature of the support increases. Low selectivity to CO_2_ is found for all mesostructured systems. In this sense, it has been widely accepted that CO_2_ formation during FT synthesis arises from both the water–gas-shift reaction (WGS) as well as from the disproportionation of carbon monoxide into CO_2_ and graphite (Boudouard reaction).^30,31^ Therefore, it can be concluded that the prepared mesostructured catalysts would show very low activity in side reactions such as WGS and Boudouard.

**Figure 11:**
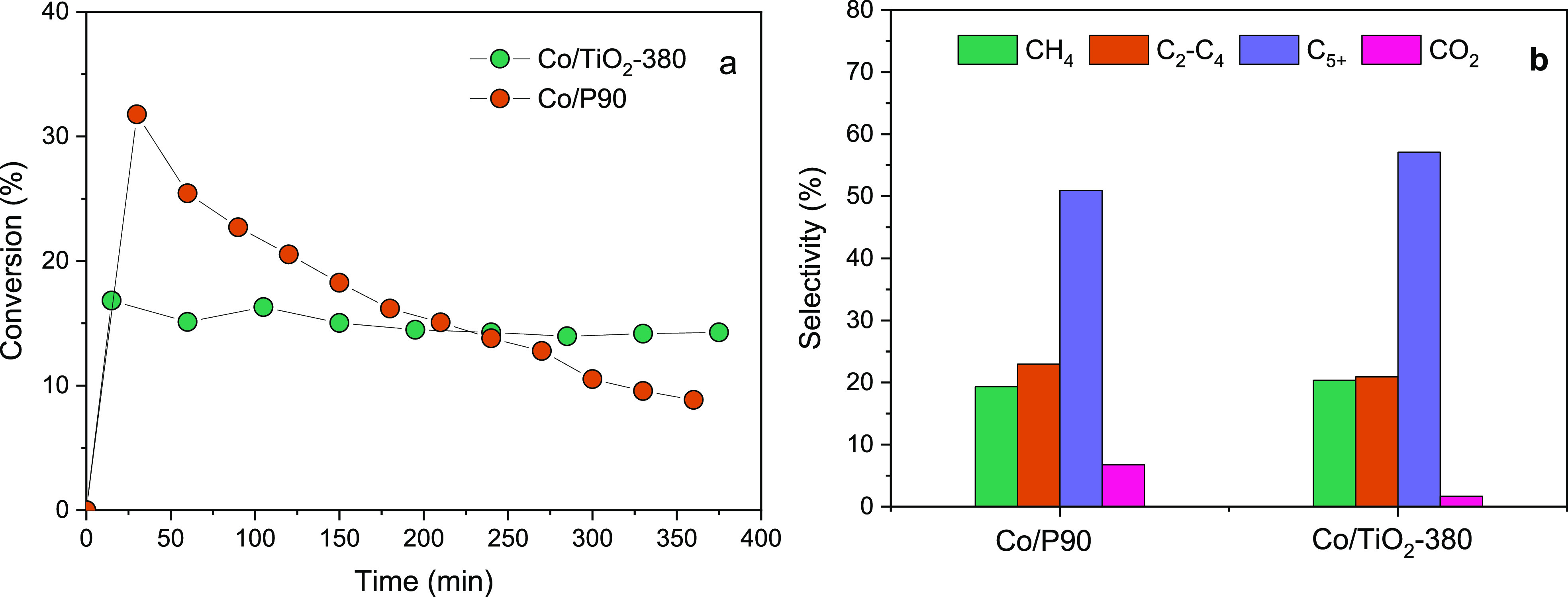
(a) CO conversion rate and (b) products selectivity during FTS reaction for Co/TiO_2_-380 and Co/P90 catalysts. Constant reaction conditions: 0.25 g of catalyst diluted in 0.25 g of SiC, *T* = 260 °C *P* = 1 MPa, GHSV = 4200 h^–1^, CO:H_2_:N_2_ = 1:2:2.

Figure 11 shows the comparison of the catalytic performance between the mesoporous Co/TiO_2_-380 and the commercial Co/P90 reference. It is observed that Co/P90 exhibits an initial CO conversion rate slightly higher than mesostructured Co/TiO_2_-380. However, after the first 30 min, the reference catalyst shows a drastic and progressive deactivation along the whole time on-stream, obtaining a final conversion of 10%, without reaching a stationary state. As we have noted before, the Co/TiO_2_-380 catalyst immediately reaches a stable pseudosteady state that is maintained over time. This behavior causes that after 6 h on-stream the mesostructured system exhibits a conversion rate of 15% (vs ca. 10% showed by Co/P90 and decaying).

Regarding the obtained product fractions, the mesostructured catalysts present a majority selectivity toward C_5+_ (ca. 60%), while the selectivity to heavy hydrocarbons in the commercial catalyst is around 50%. Almost null formation of CO_2_ is observed for the mesoporous system, while a small fraction is obtained for the commercial reference catalyst. Moreover, we have stated that C_5+_ formation reaches higher selectivity after 1 h in the case of Co/P90, coinciding with the initial drastic decay in conversion (Figure S4). C_5+_ selectivity is almost constant throughout the whole reaction period for Co/TiO_2_-380. Therefore, Co/TiO_2_-380 shows notably higher stability, with final higher conversion rates and higher C_5+_ selectivity in comparison to the Co/P90 catalyst.

**Figure 12:**
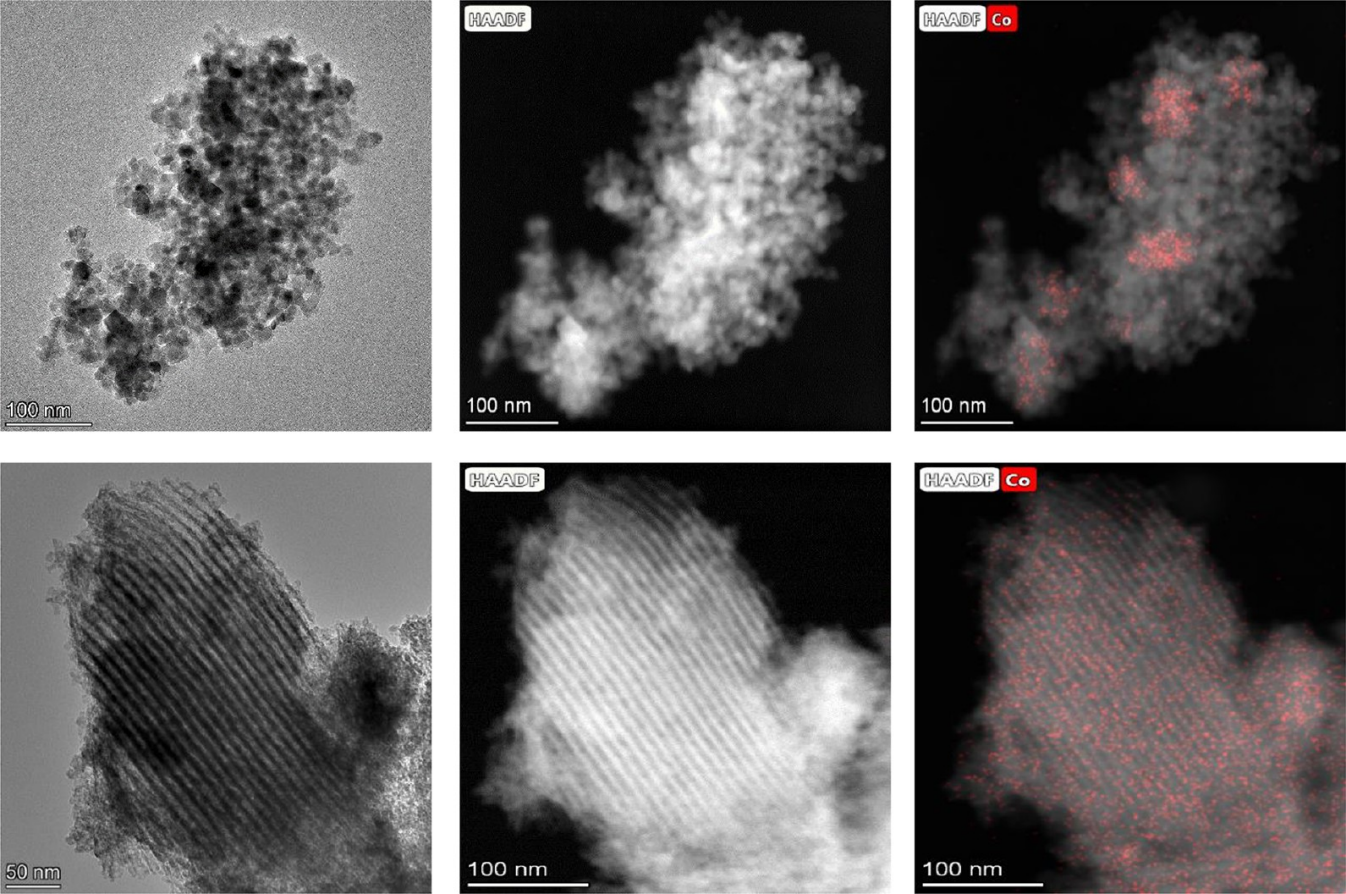
HAADF-STEM images for Co/P90 (upper row) and Co/TiO_2_-380 (lower row) after reduction at 260 °C.

**Figure 13:**
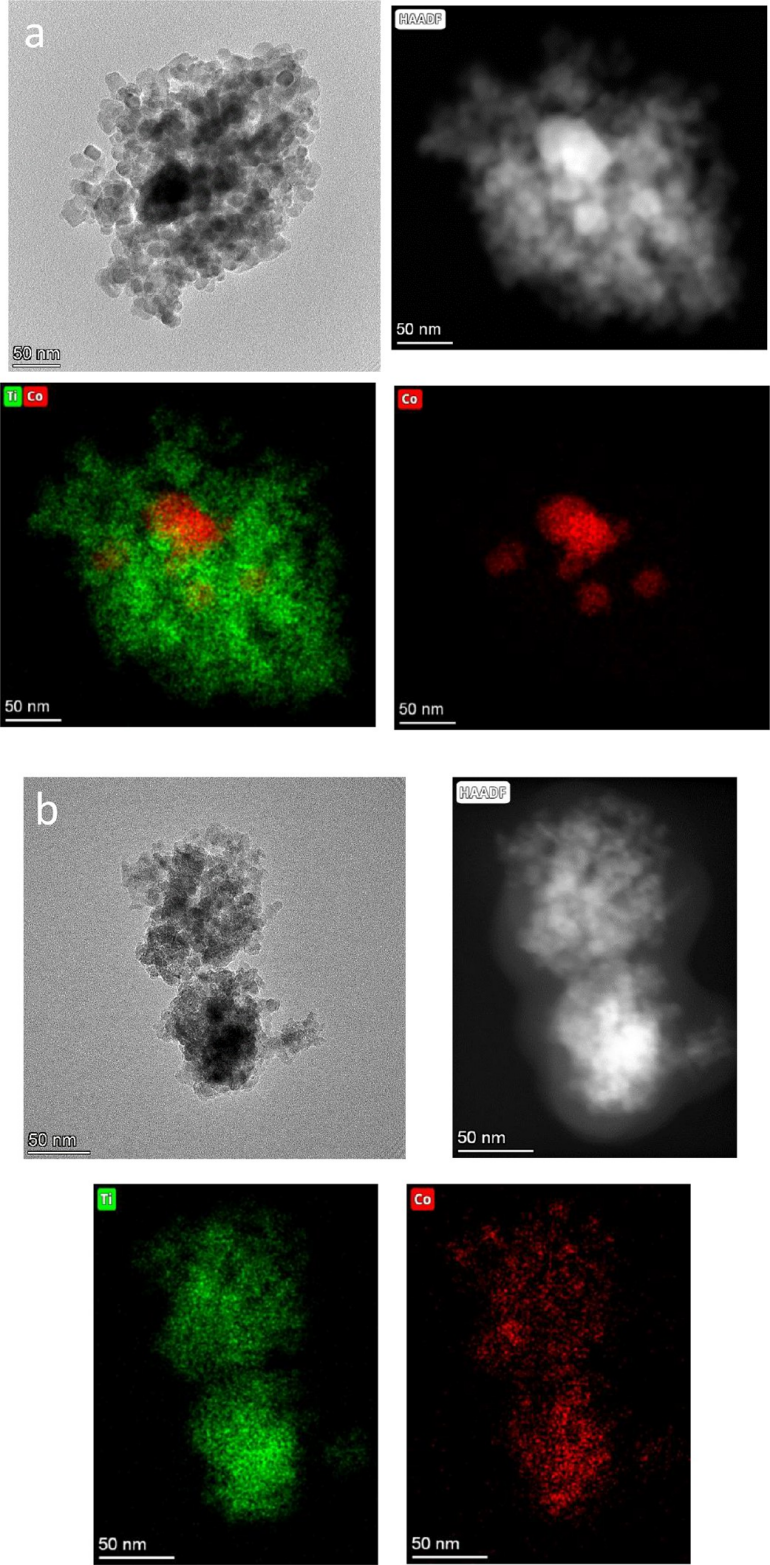
HAADF-STEM images for (a) Co/P90 and (b) Co/TiO_2_-380 catalysts after FTS reaction at 260 °C.

In order to understand the different behavior shown by mesostructured Co/TiO_2_ catalyst, we have studied by TEM the evolution of catalyst during reduction and reaction (Figures 12 and 13). Thus, as we have stated from XPS and TPR experiments, in the case of Co/TiO_2_-380 reduction treatment, it induces a significant diffusion of surface Co species toward the inner surface. In fact, by observing the TEM image for reduced Co/TiO_2_-380, it seems that Co mobilization inside the pores creates more discrete clusters along the mesoporous structure (Figure 12).

Surprisingly, the catalyst structure drastically changes after reaction. Mesoporous channels are missing so we may induce that the porous structure has been collapsed during the reaction (Figure 13a). Such severe structural modification in the mesoporous catalyst could be due to the pressure used for the reaction that would destabilize the mesoporous structure and provoke the collapse. Furthermore, cobalt nanoparticles appear to be homogeneously distributed on the support. This is an important change with respect to the Co/P90 catalyst (Figure 13b).

From N_2_ physisorption of the postreaction catalyst, we clearly confirm the collapse of the mesoporous network under reaction conditions. A BET surface area of 41 m^2^/g was observed after the reaction, which represents a loss of 75% of the specific surface area of the catalyst. In addition, the pore size was shifted from 7 to 5.5 nm, and there was a drastic decrease in the *dv/dD* value (Figure S5). This data clearly confirm the collapse of the original 7 nm pores.

We have also performed the reaction at 300 °C which corroborates the progressive collapse of the porous structure under harder reaction conditions. Thus, specific surface area drastically decreases under these reaction conditions to 18 m^2^/g, showing also an important diminution in the pore volume (Figure S5). Moreover, the postreaction catalyst shows large cobalt particles with a heterogeneous range of particle sizes, with a mean size distribution of 51 nm (Figure S6). Thus, at higher reaction temperature, the situation of Co species evolves during reaction toward a completely different situation with respect to that observed for the postreaction catalyst at 260 °C (Figure 12). Even at this temperature and in spite of the drastic surface area diminution, the catalytic performance of mesoporous systems still showed better stability with respect to the commercial catalyst (Figure S7). Thus, though a slightly higher rate is observed for the mesoporous catalyst, the progressive decay observed would denote the Co NPs agglomeration observed in Figure S6. Furthermore, a lower C_5+_ selectivity is also noticed due to the higher formation of methane.

The evolution of the Co/P90 catalyst from a fresh situation to after the reaction is different. After reduction, large Co aggregates of ca. 40–50 nm can be envisaged (Figure 11), denoting certain aggregation from the fresh situation. Then, after the reaction, important changes can be observed. In addition to these large cobalt aggregates, smaller particles around 20 nm are noticed (Figure 13). It is worth noting that these smaller particles appear completely covered by titanium oxide. Larger particles are partially covered, and so, some Co remain exposed. This fact clearly denotes an evident SMSI effect that has occurred during reaction. In this process, partially reduced TiO_*x*_ species would migrate on top of the Co^0^ particles, partially covering them and thus suppressing their H_2_ and CO chemisorption capacity.^32,33^ This behavior would explain the observed drastic and constant deactivation of the catalyst during the reactivity. Some Co particles have the opportunity to migrate, forming larger entities.

These larger NP would be the active phase, while the smaller particles that are buried in the support would become inactive. As the time on-stream increases, the largest NPs are partially covered by titanium oxide, causing a constant deactivation of the system. It is worth mentioning that this detrimental effect is avoided in the mesostructured catalyst in which the Co NPs are confined during reduction and further stabilized on the support surface during the reaction. This difference in the behavior of the catalytic systems causes their different stability in reactivity. In this way, the mesostructured system would achieve a notable improvement in activity at long reaction times.

## 4 Conclusions

We have prepared different TiO_2_ mesostructured systems by sol–gel precipitation using P-123 as the structure directing agent. After impregnation, we achieved well-dispersed Co clusters along the mesopore channels only for TiO_2_ calcined at 380 °C. For the support calcined at lower temperatures, Co species appeared widespread distributed particularly at the external surface. During the reduction treatment, an interesting cobalt diffusion process has been observed. A fraction of surface cobalt diffuses toward the inner surface forming more discrete Co clusters inside the pores and get reduced. During the reaction, the mesopore structure clearly collapses, and this confinement of reduced Co species would finally form homogeneous distribution of cobalt that clearly hinders the observed SMSI for commercial P90. Therefore, initial mesostructured TiO_2_ serves as metal NP confinement that would avoid the metal being buried due to SMSI and the progressive loss of activity. Though final TiO_2_ shows lower surface area, the well-dispersed Co NP remains unaffected by the support coverage and keeps the activity during the reaction. The outstanding stability showed by the mesoporous catalyst could be related to the observed morphological features of Co species after reduction and reaction. Thus, the use of a mesoporous TiO_2_ support leads to an almost constant rate and a higher selectivity to C_5+_.

## Supporting Information Available

HAADF-STEM image of Co/P90. Raman spectra for mesoporous Co/TiO_2_. H_2_-TPR experiments for catalysts after reduction treatment. Evolution of product selectivities during FTS reaction. Pore size distribution and HAADF-STEM images for fresh and spent Co/TiO_2_-380 catalyst. CO conversion rate and product selectivities during FTS reaction at 300 °C. (PDF)
