# Supplementary material for: Cobalt Stabilization through Mesopore Confinement on TiO2 Support for Fischer–Tropsch Reaction
Source: ACS Appl Energy Mater. 2023 Sep 8;6(18):9475–86. doi: 10.1021/acsaem.3c01432 (PMC10523356; doi:10.1021/acsaem.3c01432)
Supplement: Supplementary file 1 — ae3c01432_si_001.pdf [file ae3c01432_si_001.pdf]

Supporting Information

Cobalt Stabilization through Mesopore Confinement on TiO_2 Support for Fischer-Tropsch Reaction

F. Platero[1], S. Todorova[2], L. Aoudjera[3], L. Michelin[4,5], B. Lebeau[4,5], J.L. Blin[3], J.P. Holgado[1], A. Caballero[1], G. Colón[1*]

[1] *Instituto de Ciencia de Materiales de Sevilla. Centro Mixto Universidad de Sevilla-CSIC. Américo Vespucio, 49. 41092 Sevilla. Spain*

[2] *Institute of Catalysis, Bulgarian Academy of Sciences. 1113 Sofia, Bulgaria*

[3] *Université de Lorraine/CNRS, L2CM, UMR7053. 54500 Vandoeuvre-lès-Nancy. France*

[4] *Université de Haute Alsace, CNRS, IS2M UMR 7361, 68100 Mulhouse, France*

[5] *Université de Strasbourg, 67000 Strasbourg, France*

[*] **Corresponding Author**: gcolon@icmse.csic.es

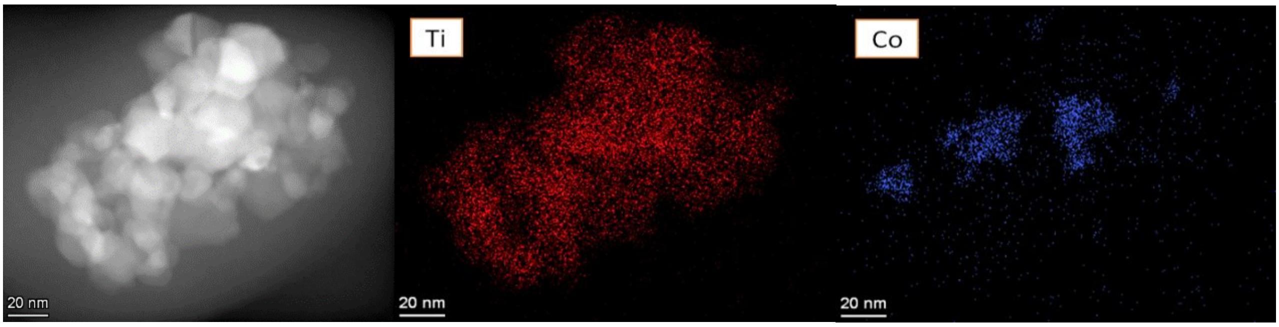

Figure S1. HAADF-STEM images for Co/P90.

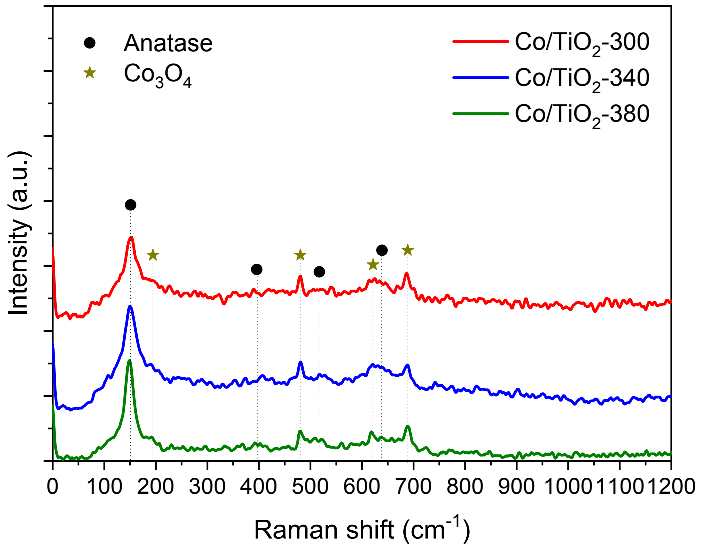

Figure S2. Raman spectra for mesoporous Co/TiO_2 catalysts.

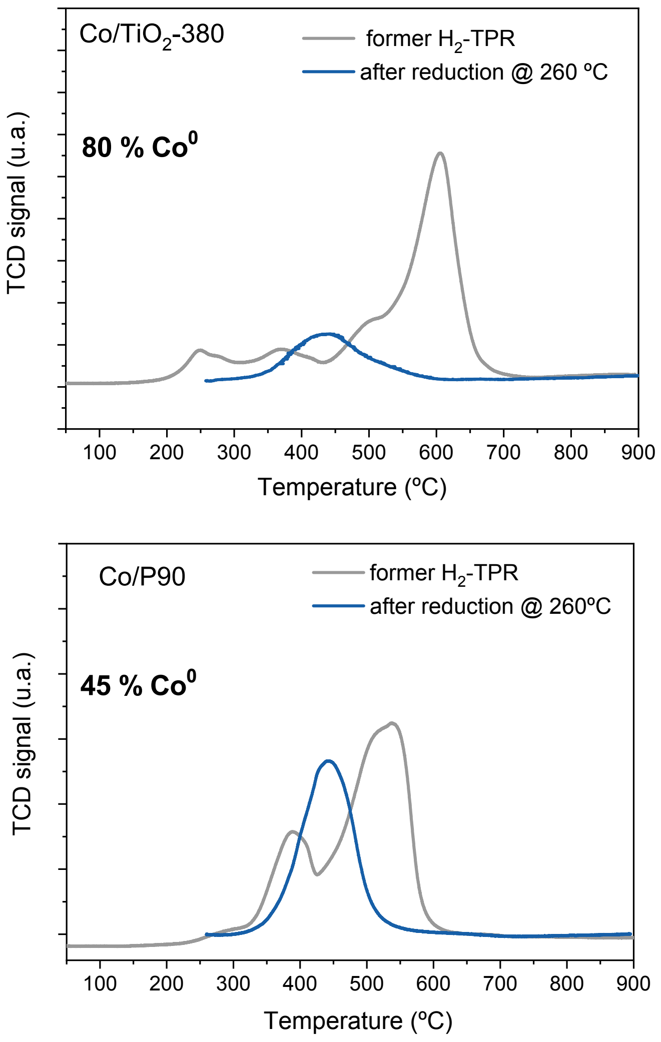

Figure S3. H_2-TPR experiments for Co/TiO_2-380 and Co/P90 after reduction treatment at 260 ºC for 13 h.

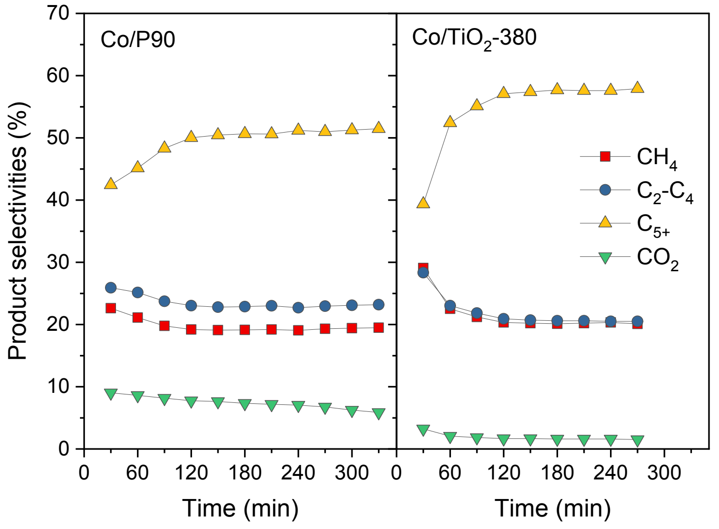

Figure S4. Evolution of product selectivities during FTS reaction at 260 ºC for Co/P90 and Co/TiO_2-380 catalysts.

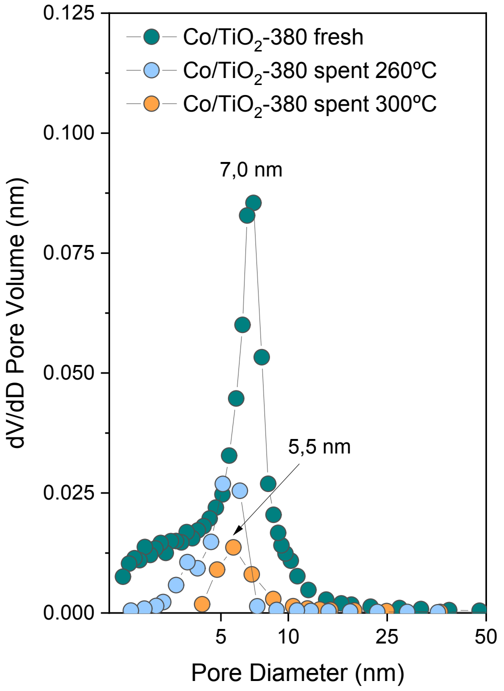

Figure S5. Pore size distribution for fresh and spent Co/TiO_2-380 catalyst.

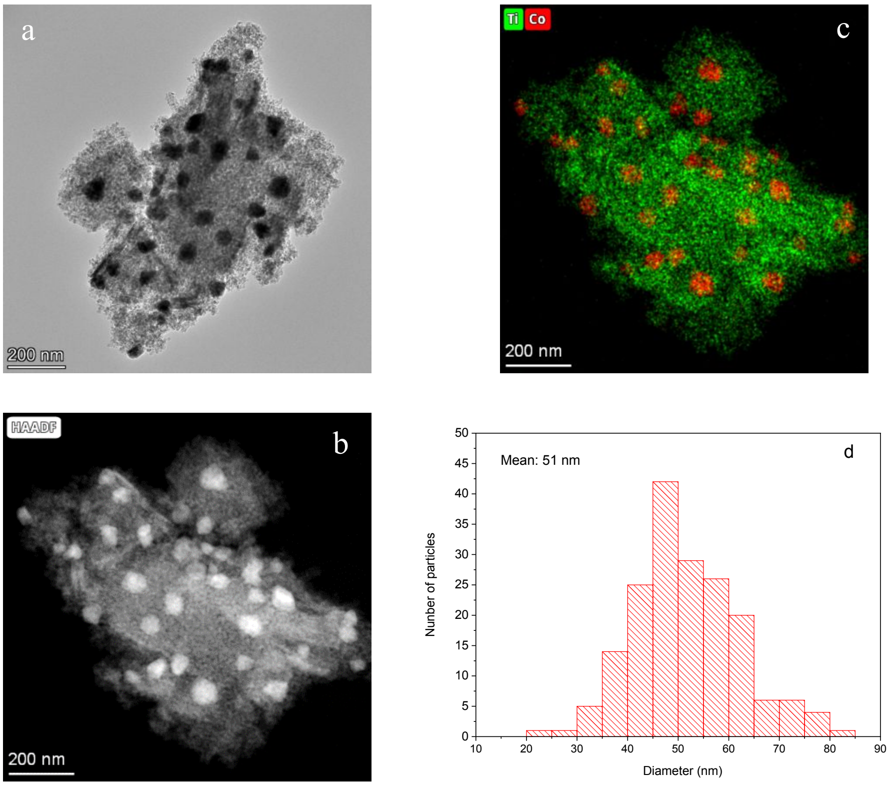

Figure S6. a-c) HAADF-STEM images and d) particle size distribution for Co/TiO_2-380 catalyst after FTS reaction at 300 ºC.

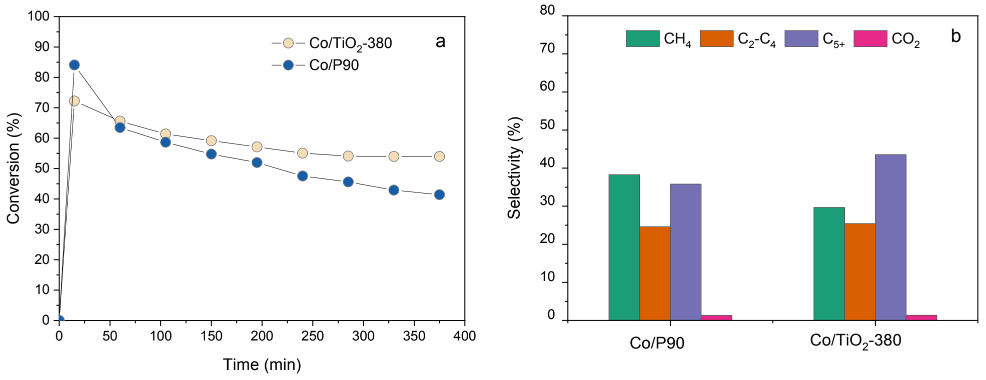

Figure S7. CO conversion rate and product selectivities for Co/TiO_2-380 and Co/P90 catalysts during FTS reaction at 300 ºC.
